# Clinical resolution of oral lichenoid lesions after amalgam replacement: A systematic review and meta-analysis of observational studies

**DOI:** 10.1016/j.jobcr.2026.01.008

**Published:** 2026-01-28

**Authors:** Harshita Kothari, Ajinkya M. Pawar, Pankaj Gupta, Alexander Maniangat Luke, Mohamed Saleh Hamad Ingafou, Parmeet Banga, Mohmed Isaqali Karobari, Dian Agustin Wahjuningrum

**Affiliations:** a Department of Conservative Dentistry and Endodontics, Nair Hospital Dental College, Affiliated to Maharashtra University of Health Sciences (MUHS), Mumbai, Maharashtra, India; b Department of Clinical Science, College of Dentistry, Ajman University, Al-Jruf, Ajman, United Arab Emirates; c Centre of Medical and Bio-Allied Health Science Research, Ajman University, Al-Jruf, Ajman, United Arab Emirates; d Department of Prosthodontics, YMT Dr. G.D. Pol Foundation Y.M.T Dental College and Hospital, Kharghar, Navi Mumbai, Maharashtra, India; e Department of Restorative Dentistry & Endodontics, Faculty of Dentistry, University of Puthisastra, Phnom Penh, 12211, Cambodia; f Department of Conservative Dentistry, Faculty of Dental Medicine, Universitas Airlangga, Jawa Timur, Indonesia; g Research Center for Biomaterial and Tissue Engineering, Faculty of Dental Medicine, Universitas Airlangga, Indonesia

**Keywords:** Biocompatible materials, Dental amalgam, Hypersensitivity reaction, Oral lichenoid lesions, Oral mucosa

## Abstract

**Introduction:**

Oral lichenoid lesions (OLLs) can mimic oral lichen planus but are often linked to contact with dental amalgam. Replacement with biocompatible materials has been associated with lesion resolution, yet prior evidence lacked quantitative synthesis.

**Materials and methods:**

Following PRISMA 2020 (PROSPERO: CRDXXXXXXXXXX2), eight databases (1986–2024) were searched for adult in vivo studies reporting OLL resolution after amalgam replacement. Two reviewers independently screened, extracted data, and assessed bias (ROBINS-I, Newcastle–Ottawa Scale, JBI). Random-effects meta-analysis estimated pooled odds ratios (OR) for clinical resolution; certainty was graded with GRADE.

**Results:**

Of 2368 records, 35 studies were qualitatively synthesized and 11 entered meta-analysis (N = 365). Amalgam was replaced with glass ionomer, ceramics, composite resin, or gold alloys. Replacement markedly increased odds of lesion resolution (OR = 122.18; 95 % CI: 55.64–268.26; p < 0.001) with moderate heterogeneity (I^2^ = 42 %). Risk of bias across observational studies was generally low to moderate. Funnel-plot asymmetry and Egger's test (p < 0.001) suggested publication bias; overall certainty was moderate. Subgroup analyses indicated greater benefit when lesions had direct amalgam contact, when patch testing was positive, and when ceramics or gold were used. Sensitivity analyses supported strength of the primary findings.

**Conclusion:**

Amalgam replacement was linked with substantially higher odds of OLL resolution. However, between-study heterogeneity and indications of publication bias temper confidence in the pooled effect. Confirmatory, prospective, registered studies with standardized diagnostic criteria, outcome definitions, and material categories are needed to refine effect size estimates and strengthen certainty.

## 1 Introduction

It is important to distinguish amalgam associated Oral lichenoid lesions (OLLs) contact lesions from idiopathic oral lichen planus in order to make a valid inference.^1^^,^^2^ Contact lesions are typically unilateral or confined to mucosa in direct contact with restorations, show no extraoral involvement, and often improve after replacement of the implicated material.^3^^,^^4^ Because histopathology can overlap with lichen planus, diagnosis relies on clinicopathologic correlation and, when feasible, elimination testing.^5^ To minimize misclassification bias, we analyzed idiopathic oral lichen planus separately from contact lesions and interpreted pooled effects accordingly.^6^

For many years, dental amalgam contains mercury, silver, tin, and copper has been used more than other restorative materials due to favourable characteristics, strength, durability, and reasonable cost. Certain individuals, particularly with Type IV hypersensitivity, may develop a localized immune-mediated mucosal Inflamed condition after a chronic exposure to amalgam.^7^ Inflammation mediated by T cells can lead to chronic oral lesions, which may be a debilitating condition by showing signs of pain, burning, and difficulty function.^8^ Because OLL and OLP can be misclassified and patch-testing has imperfect sensitivity/specificity, we emphasized clinicopathologic correlation and response to elimination when defining eligibility.^9^^,^^10^

The health literature regarding the healing ability of OLLs after amalgam replacement is inconsistent and unclear. Reports vary from completely healed lesions in weeks or months after amalgam removal to only partial or no improvement which may relate to lesion subtype, duration of exposure, the use of substitute material and the immunological status of the patient.^2^^,^^11^ The duration of follow-up is heterogeneous between studies, with poor consistency in measures of outcome preventing cross-study comparisons or development of best-practice recommendations.

Although prior reviews and case series have included reports of OLLs in association with amalgam, evidence has mainly been descriptive or anecdotal with limited formal statistical synthesis or clarity on probative factors.^12^ A high quality systematic review and meta-analysis is needed to bring together the available data to more accurately quantify the true clinical significance of amalgam removal, as well as identify and assess any patient or lesion specific factors that might affect outcomes.

OLLs affect oral function and psychological well-being, with disproportionate impact on vulnerable groups who face barriers to specialist care and may rely on lower-cost amalgam. In the context of the Minamata Convention's phase-down of mercury-containing dental materials,^13^ OLLs—potentially reflecting mucosal hypersensitivity—are a clinically relevant consequence that should inform both clinical decision-making and policy.

This systematic review and meta-analysis tests the hypothesis that replacing amalgam, compared with no replacement, increases the likelihood of complete clinical resolution of oral lichenoid lesions. The primary objective is to estimate the effect of amalgam replacement on lesion resolution or healing relative to either retaining amalgam or replacing it with alternative materials. Secondary objectives are to evaluate prognostic modifiers, specifically lesion subtype (contact lesion versus idiopathic oral lichen planus), the restorative material selected for replacement, and the proximity of the lesion to the implicated restoration, and to summarize time to improvement, adverse events, and restoration performance.

## 2 Materials and Methods

### 2.1 Study design and protocol registration

This systematic review and meta-analysis followed the Preferred Reporting Items for Systematic Reviews and Meta-Analyses (PRISMA 2020) standards.^14^ The study protocol is registered with the International Prospective Register of Systematic Reviews (PROSPERO) - registration number CRDXXXXXXXXXX2. The methodology aligned with the Cochrane Handbook for Systematic Reviews of Interventions (Version 5.1.0)^15^ and Joanna Briggs Institute (JBI) Reviewer's Manual.^16^

### 2.2 Eligibility criteria

The eligibility criteria were defined using the PEOS (Population, Exposure, Outcome, Study design) model.^17^

• Population: Adults diagnosed with clinically and/or histologically confirmed oral lichenoid lesions (OLLs) associated with or directly related to dental amalgam restorations.
• Exposure: Replacement of amalgam restorations with a different restorative material including composite resin, gold, ceramic, glass ionomer cement, or a combination of these materials.
• Outcome: Partial or complete clinical resolution of OLLs following amalgam replacement as noted by the authors of the studies.
• Study design: in vivo studies, including randomized controlled trials (RCTs), non-randomized clinical trials, prospective cohort studies, comparative studies, and case reports. Full-text, peer-reviewed articles were included from January 1986 to December 2024. Articles in languages other than English were to be included if a reliable translation to English was available.

### 2.3 Exclusion criteria

• In vitro studies, animal studies, case series, and abstracts without available full text.
• Non-reports of outcome data on resolution of OLL following amalgam replacement were also excluded.

### 2.4 Focused review question

The purpose of this systematic review and meta-analysis was to determine a particular study question: Does substituting dental amalgam with biocompatible dental materials enable oral lichenoid lesions in adult patients to heal?

### 2.5 Search strategy

The following databases were searched extensively for electronic literature: PubMed/MEDLINE, Cochrane CENTRAL, EMBASE, CINAHL, PsycINFO, Scopus, ERIC, and ScienceDirect. The search was language-unrestricted and included studies available through December 2024. Search terms included combinations of MeSH and free-text keywords related to PEOS. Full database-specific search strings (PubMed/MEDLINE, EMBASE, CENTRAL, CINAHL, PsycINFO, Scopus, ERIC, ScienceDirect), including field tags and thesaurus explosions, are provided in Supplementary Table S1.

### 2.6 Study selection

Following an initial screening of abstracts and titles by two independent reviewers, comprehensive full-text analyses of potentially pertinent articles were undertaken. Discrepancies were resolved through consensus, and a third reviewer was engaged when arbitration was necessary. Cohen's kappa coefficient (κ), which quantifies inter-rater agreement for study inclusion, was 0.84, signifying strong concordance.

### 2.7 Data extraction

Two reviewers independently extracted study-level and participant-level information using a piloted form. For each study, we recorded author, year, country, and design; participant age and sex; lesion characteristics (type, location, and diagnostic approach); details of the restorative intervention (material selected for replacement); outcome measures (complete or partial resolution and follow-up duration); and any declaration of funding or conflicts of interest. Disagreements were resolved by discussion or, when necessary, adjudication by a third reviewer. Patch-testing status (performed/not performed and positive/negative result) was abstracted as a predefined subgroup variable based on the a priori hypothesis that sensitization might modify healing.

### 2.8 Risk of bias and quality assessment

The risk of bias in each of the articles that contributed to this review was assessed using the following approaches:

• For non-randomized studies, the ROBINS-I tool was implemented to evaluate risk across seven domains: confounding; bias in participant selection; bias in classification of interventions; bias due to deviations from intended interventions; bias due to missing data; bias in the measurement of outcomes; and bias due to selective reporting.
• In the case of comparative cross sectional studies, the Newcastle-Ottawa Scale (NOS) which was adapted for the cross sectional design was used.
• Finally, for case reports, the Joanna Briggs Institute (JBI) Critical Appraisal Checklist for Case reports was used to evaluate clinical history; the diagnosis; any interventions; outcomes; and conclusions.

Assessment was completed by two independent assessors, with any disagreements resolved through consensus. Although case reports sit low on the evidence hierarchy, we included them to capture rare but clinically relevant presentations and to contextualize patterns observed in comparative cohorts; they contributed only to the qualitative synthesis and hypothesis generation.

### 2.9 Data synthesis and statistical analysis

We anticipated methodological and clinical heterogeneity and therefore used random-effects models to synthesize dichotomous outcomes as odds ratios (ORs) with 95 % confidence intervals. The primary endpoint was complete or partial clinical resolution following amalgam replacement versus no replacement (or retention/alternative material). Heterogeneity was quantified with I^2^ and interpreted using conventional thresholds (approximately 0–40 % low, 30–60 % moderate, 50–90 % substantial, and >75 % considerable). When sufficient data were available, we explored prespecified subgroups—lesion type, restorative material, geographic region, and follow-up duration—and performed sensitivity analyses that sequentially omitted individual studies and restricted to lower risk-of-bias evidence. Planned meta-regression for lesion site, age, and replacement material was contingent on data sufficiency and was not pursued where strata were sparse. Publication bias and small-study effects were assessed using funnel plot visualization, Egger's regression test, and additionally the Doi Plot with Luis–Furuya-Kanamori (LFK) Index, which provides greater sensitivity for asymmetry detection in sparse binary event meta-analyses. All analyses were conducted in Comprehensive Meta-Analysis (v3) and RevMan (v5.4).

## 3 Results

**Fig. 1:**
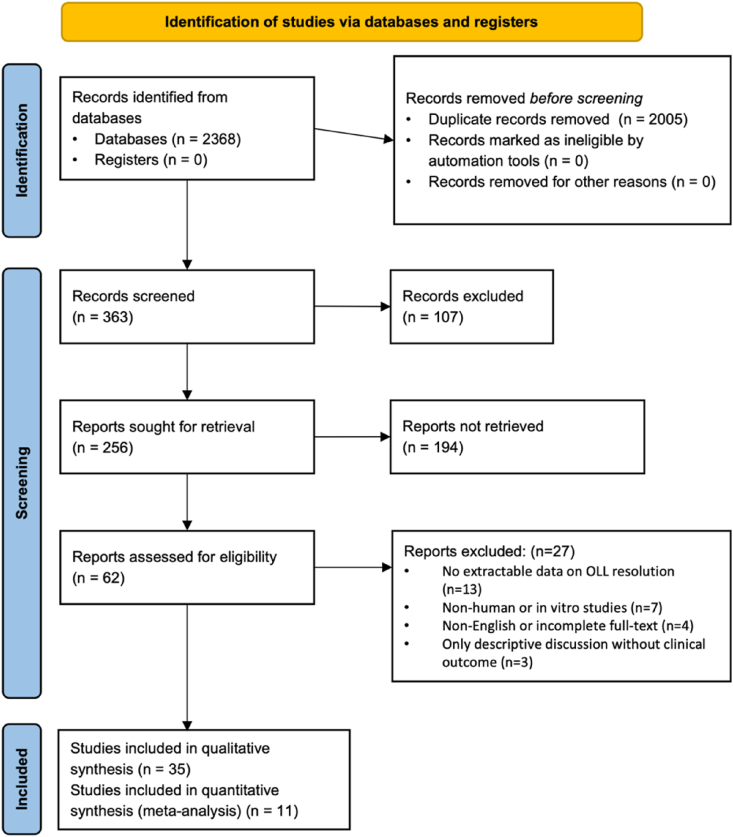
PRISMA 2020 study selection flow diagram.

A systematic search of eight databases resulted in 2368 records. After 2005 duplicates were removed, 363 titles and abstracts were screened. Following our inclusion and exclusion criteria, 62 full-text articles were reviewed, and 35 studies were deemed eligible for qualitative synthesis. Database coverage and pre-deduplication yields are summarized in Table 1; complete Boolean strategies appear in Supplementary Table S1. Of those, 11 studies had data for extractable outcomes and were included in the quantitative meta-analysis. The study selection process is summarized in the PRISMA 2020 flow diagram (Fig. 1).

**Table 1:** Overall Search strategy in PubMed.

Search strategy	No. of articles obtained
((((((("dental amalgam"[MeSH Terms] OR ("dental"[All Fields] AND "amalgam"[All Fields]) OR "dental amalgam"[All Fields] OR "amalgam"[All Fields]) AND restorations[All Fields]) OR (("mercury"[MeSH Terms] OR "mercury"[All Fields]) AND ("hypersensitivity"[MeSH Terms] OR "hypersensitivity"[All Fields] OR "allergy"[All Fields] OR "allergy and immunology"[MeSH Terms] OR ("allergy"[All Fields] AND "immunology"[All Fields]) OR "allergy and immunology"[All Fields]))) AND (("mouth"[MeSH Terms] OR "mouth"[All Fields] OR "oral"[All Fields]) AND lichenoid[All Fields] AND lesions[All Fields])) OR ("lichen planus, oral"[MeSH Terms] OR ("lichen"[All Fields] AND "planus"[All Fields] AND "oral"[All Fields]) OR "oral lichen planus"[All Fields] OR ("oral"[All Fields] AND "lichen"[All Fields] AND "planus"[All Fields]))) OR ("dermatitis, allergic contact"[MeSH Terms] OR ("dermatitis"[All Fields] AND "allergic"[All Fields] AND "contact"[All Fields]) OR "allergic contact dermatitis"[All Fields] OR ("contact"[All Fields] AND "allergy"[All Fields]) OR "contact allergy"[All Fields])) AND (((((("dental amalgam"[MeSH Terms] OR ("dental"[All Fields] AND "amalgam"[All Fields]) OR "dental amalgam"[All Fields] OR "amalgam"[All Fields]) AND restoration[All Fields] OR "replacement"[All Fields])) OR (composite[All Fields] AND restorations[All Fields])) OR (("gold"[MeSH Terms] OR "gold"[All Fields]) AND restorations[All Fields])) OR ("glass ionomer cements"[MeSH Terms] OR ("glass"[All Fields] AND "ionomer"[All Fields] AND "cements"[All Fields]) OR "glass ionomer cements"[All Fields] OR ("glass"[All Fields] AND "ionomer"[All Fields] AND "cement"[All Fields]) OR "glass ionomer cement"[All Fields])) OR ("dental porcelain"[MeSH Terms] OR ("dental"[All Fields] AND "porcelain"[All Fields]) OR "dental porcelain"[All Fields] OR "porcelain"[All Fields]))) AND (("wound healing"[MeSH Terms] OR ("wound"[All Fields] AND "healing"[All Fields]) OR "wound healing"[All Fields] OR "healing"[All Fields]) AND lichenoid[All Fields] AND lesions[All Fields])	62

### 3.1 Characteristics of the included clinical studies

A total of 19 clinical studies (17 prospective and 2 comparative) were included. The studies in this analysis examine multiple healthcare settings and populations and are located in different parts of the world, such as Europe 18, 19, 20, 21, 22, 23, 24, 25, 26, 27, 28, 29, 30, 31, Asia 32, 33, 34, and Australia.^35^^,^^36^ Most of the participants in the clinical studies were female and ranged in age from 24 to 78. All cases showed a unilateral distribution and a direct topographic association with amalgam restorations, with the majority of lesions (85 %) found on the buccal mucosa. An overview of the key findings from the prospective and comparative studies is given in Table 2.

**Table 2:** Characteristics of included clinical studies (prospective: 17 studies and comparative: 2 studies designs).

Study ID	Country	Study Design	Sample Size (Age)	Lesion Type	Lesion Location	Diagnostic Methods	Restorative Material Used	Intervention	Follow-up Duration	Reported Outcome
Lind, 1988^18^	Finland	Prospective	n = 52 (24–68 yrs)	Erosive OLL	Buccal mucosa, tongue, gingiva	Biopsy, patch test	Composite, gold, GIC	Full replacement	20 months–6 yrs	89 % complete remission
Laine, 1991^19^	Norway	Prospective	n = 52 (24–68 yrs)	Erosive OLL	Buccal mucosa	Biopsy	Composite, gold, porcelain	Full replacement	4–36 months	89 % complete resolution
Henriksson, 1995^20^	Sweden	Prospective	n = 159	White, red, ulcerated	Molar-retromolar	Patch test (few cases)	Not reported	Amalgam removal (n = 131)	Not stated	92 % improved/healed
Pang, 1995^35^	Australia	Prospective	n = 41 (28–72 yrs)	Reticular, erosive, plaque	Buccal mucosa, tongue, gingiva	Patch test	Composite, gold, porcelain	Partial replacement (n = 16)	2 months–9.5 yrs	All improved/resolved
Östman, 1995^21^	Sweden	Prospective	n = 49 (34–78 yrs)	Reticular/papular	Contact & beyond contact	Patch test	Composite, gold, porcelain	Full (84 %) or partial (16 %)	3 months–4 yrs	69 % clinical, 55 % histological resolution
Bratel, 1996^22^	Sweden	Prospective	n = 142 OLR, 19 OLP	Reticular, plaque	Oral mucosa	Repeat biopsy	Gold, metal-ceramic	Full replacement	Not stated	95 % improved/resolved
Dunsche, 2003^23^	Germany	Prospective	n = 134 (22–72 yrs)	Reticular, plaque, erosive	Buccal mucosa, tongue	Biopsy, patch test	Composite, gold, GIC, titanium	Full (n = 92) or partial (n = 13)	Up to 34 months	29.5 % full, 60 % partial improvement
Thornhill, 2003^24^	UK	Comparative	n = 81 (avg. 54.6 yrs)	Reticular, plaque, erosive	Oral mucosa	Patch test	Composite, gold, GIC, ceramic	Full replacement (n = 28)	3–12 months	71.4 % full, 21.4 % partial resolution
Wong, 2003^36^	Australia	Prospective	n = 84 (28–84 yrs)	Reticular, plaque, erosive, atrophic	Buccal mucosa, tongue, gingiva	Patch test	Composite, gold, porcelain	Replacement (n = 30)	2 months–9.5 yrs	93 % improved; 90 % resolved
Laeijendecker, 2004^25^	Netherlands	Prospective	n = 60	Hyperkeratotic, erosive	Not reported	Patch test, biopsy	Not reported	Replacement in patch-positive cases	3 months	Improvement in contact-based cases
Issa, 2005^26^	UK	Prospective	n = 51 (avg. 53 yrs)	Reticular, erosive	Buccal mucosa, tongue	Patch test	Composite, gold, ceramic	Full replacement (n = 39)	4–32 months	42 % resolved, 47 % improved
Pezelj-Ribarić, 2008^27^	Croatia	Prospective	n = 20 (OLR)	Reticular, erosive	Buccal mucosa, tongue, gingiva	Biopsy, patch test	Composite, gold, porcelain	Full replacement	2 months–3.5 yrs	80 % resolved, 15 % improved
Lartitegui-Sebastian, 2012^28^	Spain	Prospective	n = 100 (7 OLL cases)	White macules, erosive	Buccal and lingual mucosa	Patch test	Composite	Full replacement	3–6 months	71.4 % improved, 14.3 % resolved
Montebugnoli, 2012^29^	Italy	Prospective	n = 64	Reticular, bilateral	Oral mucosa	Patch test	Composite	Full replacement	3 months–2 yrs	22 % complete regression
Mårell, 2014^30^	Norway	Prospective	n = 44 (37–78 yrs)	Not specified	Oral mucosa	Biopsy	Not reported	Full/partial replacement	Not specified	71 % remission in LCR vs. 8 % OLP
Thanyavuthi, 2016^32^	Thailand	Prospective	n = 53 (avg. 50.8 yrs)	Reticular, erosive, ulcerative	Buccal mucosa, tongue	Patch test	Not specified	Recommended replacement	6 months	30 % resolved, 70 % significant improvement
Karatasli, 2018^31^	Turkey	Prospective	n = 24 (avg. 45 yrs)	Reticular, plaque, erosive	Buccal mucosa	Biopsy, patch test	Feldspathic ceramic	Full replacement (n = 16)	3 months–5 yrs	63 % resolved, 31 % improved
Gupta, 2022^33^	India	Prospective	n = 60 (OLCLs/OLP)	Reticular	Tongue, buccal mucosa	Not reported	Not reported	Not reported	Not reported	March 2000 patients showed full resolution
Tsushima, 2022^34^	Japan	Comparative	n = 41	Not reported	Buccal mucosa, gingiva, tongue	Patch test	Composite, ceramic	Metal removal	Not reported	95.8 % full or partial resolution

### 3.2 Characteristics of the included case reports

Additionally, 16 case reports 37, 38, 39, 40, 41, 42, 43, 44, 45, 46, 47, 48, 49, 50, 51, 52 were added to provide practical, patient-centered data to support the clinical evidence. These papers focused on lesion resolution after amalgam replacement, diagnostic problems, and patch testing results. Most instances showed full or almost full clinical improvement after switching to biocompatible restorative materials such glass ionomer cement or composite resin. Table 3 presents an overview of these reports.

**Table 3:** Characteristics of included case reports on amalgam-associated oral lichenoid lesions (OLLs).

Author (Year)	Age/Sex	Chief Complaint	Lesion Site	Restoration	Diagnostic Test	Replacement Material	Outcome
Sunith (2006)^37^	54/F	Burning sensation	R buccal mucosa	Amalgam (tooth 16)	Patch test	GIC	Healed
Kal (2008)^38^	20/F	Burning/itching	R buccal mucosa	Mandibular molars	Patch test	Temp filling	Immediate symptom relief
Aggarwal (2010)^39^	23/M	Sensitivity, discomfort	Bilateral buccal	Bilateral molars	Biopsy, patch test	Interim restoration	Healed
Barbosa (2011)^40^	52/F, 76/F	Burning, irritation	Tongue, buccal mucosa	Posterior molars	Not stated	Composite	Healed
Kharangate (2013)^41^	26/M	Burning	L buccal mucosa	Tooth 27	Patch test	GIC	Resolved
Ramnarayan (2014)^42^	30/F	Black patch, discomfort	R buccal mucosa	Teeth 16, 46	Histology	Composite	Healed
Dahiwal (2014)^43^	40/F, 28/F	Pain, burning	Buccal mucosa	Teeth 36, 47	Not done	Composite	Healed
Shalabh (2014)^44^	35/F	Pain on eating	Bilateral buccal	Multiple posterior	Not stated	GIC → Composite	Resolved
Jyothi (2015)^45^	34/F, 59/F, 45/F	Burning	Buccal mucosa	Multiple molars	Not stated	Composite	Resolved
Marouane (2015)^46^	26/F	Burning, erosion	R buccal mucosa	Tooth 46	Patch test	Composite	Healed
Pawar (2016)^47^	42/M	Bilateral burning	Buccal mucosa	Teeth 36, 47	Patch test	GIC	Significant improvement
Sarraf (2017)^48^	48/F	Pain with spicy food	L buccal mucosa	Teeth 36, 38	Patch test	ZOE interim	Resolved
Guvenc (2019)^49^	37/M	Discomfort	Mand. cheek	Tooth 46	Biopsy	Not specified	Symptom reduction
Klasiri (2019)^50^	67/F	Soreness	L buccal mucosa	Teeth 28, 33	Not stated	Composite	Healed
Petruzzi (2022)^51^	42/F	Burning, itching	Tongue (lateral)	Teeth 36, 37	Patch test	Composite	Resolved
Bettoni (2023)^52^	61/F	White lesions	Dorsal tongue	Teeth 25, 26	Biopsy	Zirconia crown	Resolved

### 3.3 Diagnosis, intervention, and follow-up characteristics

Lesions were classified as reticular (48 %), erosive (29 %), and plaque-like (23 %). All studies confirmed a topographic relationship between the amalgam restoration and lesion site. Diagnosis was primarily clinical, supported by histopathology (70 %) and patch testing (performed in 66 %, with positive results in 58 % of those tested). Amalgam restorations were replaced with composite resin (52 %), gold (18 %), feldspathic ceramic (12 %), or glass ionomer cement (GIC; 8 %), depending on availability and clinical context. Follow-up periods ranged from 3 to 24 months. The specifics of the diagnosis procedures and intervention characteristics are listed in Table 4.

**Table 4:** Diagnostic approaches and restorative interventions for OLLs across study types.

Parameter	Prospective Clinical Trials (n = 16)	Comparative Studies (n = 3)	Case Reports (n = 16)
**Diagnostic Confirmation Methods**	- Clinical exam: 100 % - Histopathology: 81 % - Patch testing: 69 %	- Clinical exam: 100 % - Histopathology: 67 % - Patch testing: 100 %	- Clinical exam: 100 % - Histopathology: 58 % - Patch testing: 53 %
**Patch Test Positivity**	Reported in 11 studies; positive in 7	Positive in all 3 studies	Reported in 10 studies; positive in 6
**Type of Material Used for Replacement**	- Composite resin: 10 studies - Gold: 3 - Ceramic: 2 - GIC: 1	- Composite resin: 2 studies - Gold: 1	- Composite resin: 10 - Gold: 4 - Ceramic/GIC: 5
**Restorative Technique**	Direct restoration replacement under rubber dam isolation	Standardized direct technique	Conventional replacement, mostly non-standardized
**Follow-Up Duration**	6–24 months (mean: ∼12 months)	6–12 months	3–18 months (variable)
**Monitoring of Healing**	Clinical exam + photographic documentation + patient-reported symptoms	Clinical evaluation + scoring of lesion resolution	Mainly clinical exam and patient feedback
**Reported Healing Outcome**	Full or partial resolution in all 16 studies	All 3 reported significant resolution post replacement	17 of 19 reported complete or near-complete resolution

### 3.4 Risk of bias assessment

**Fig. 2:**
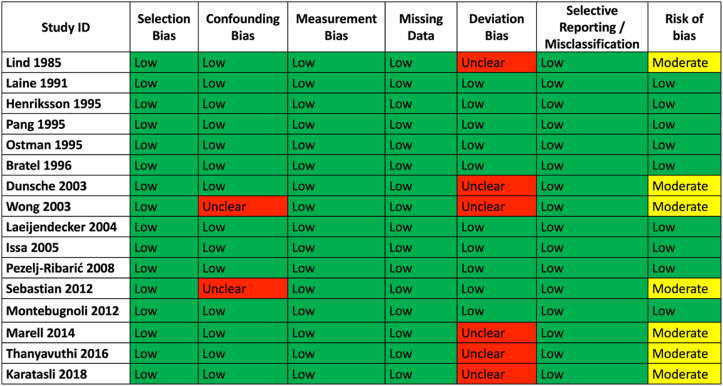
Risk of Bias assessment for included non-randomized studies.

When considering the non-randomized studies (Fig. 2), nine of the sixteen had low risk of bias 19, 20, 21, 22^,^^25^, ^26^, ^27^^,^^29^^,^^35^, while the others were judged to be at moderate risk ^18^^,^^23^^,^^28^^,^^30^, ^31^, ^32^^,^^36^. In the moderate risk studies, this was primarily due to insufficient information on the fidelity of the intervention and the controls for confounding.

In the class of comparative studies, all three studies^24^^,^^33^^,^^34^ scored ≥5 on the Newcastle-Ottawa Scale indicating fairly high levels of methodological quality (Table 5).

**Table 5:** Newcastle-Ottawa Scale (NOS) Assessment of included comparative studies.

Study ID	Selection				Comparability		Outcome		Total Score (★/9)
	Sample Representativeness	Sample Size	No-response	Determination of Exposure	Primary Factor	Additional Consideration	Outcome Evaluation	Statistical Testing	
Thornhill et al., 2003^24^	★	–	–	★	★	–	★	★	5
Gupta et al., 2022^33^	★	–	★	★	★	–	★	★	6
Tsushima et al., 2022^34^	★	–	★	★	★	–	★	★	6

**Fig. 3:**
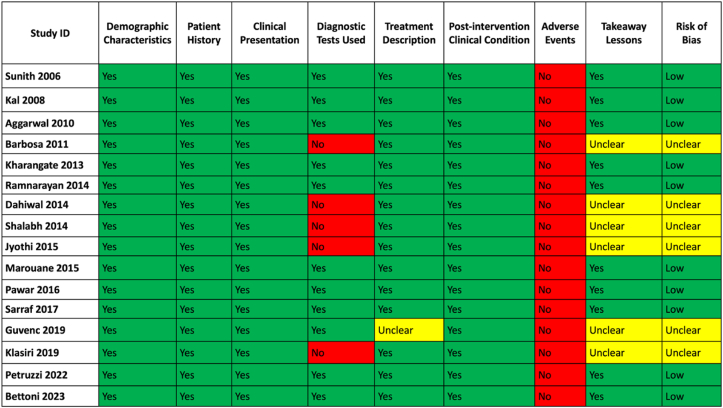
Case report quality assessment.

Regarding case reports, ten 37, 38, 39^,^^41^^,^^42^^,^^46^, ^47^, ^48^^,^^51^^,^^52^ out of 16 demonstrated a low risk of bias, although none reported adverse events (Fig. 3). All included case reports documented clinical improvement following amalgam replacement. Overall, the quality of evidence for the included quantitative studies was assessed as moderate to high.

### 3.5 Meta-analysis of lesion resolution post-amalgam replacement

**Fig. 4:**
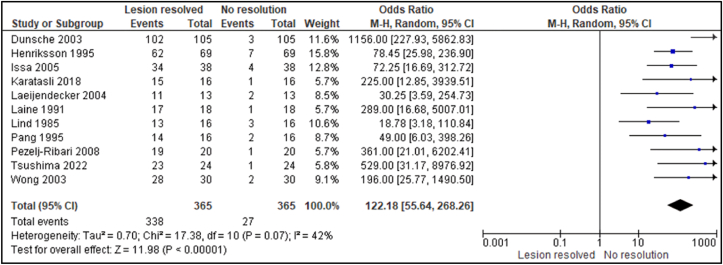
A forest plot illustrating the odds ratio (OR) with 95 % confidence intervals (CI) for the comparison of lesion resolution against non-resolution across various studies.

The studies included in the quantitative synthesis comprised ten prospective non-randomized clinical studies 18, 19, 20^,^^23^^,^^25^, ^26^, ^27^^,^^31^^,^^35^^,^^36^ and one comparative observational study,^34^ all of which reported extractable dichotomous outcomes describing complete or partial lesion resolution following amalgam replacement. These were considered sufficiently compatible for quantitative synthesis because all studies evaluated the same exposure (amalgam replacement), applied comparable clinical outcome definitions (complete or partial lesion resolution), and reported extractable dichotomous data with clearly defined pre- and post-intervention measurements. Importantly, none of the included studies involved randomization or complex co-interventions; thus, the comparative study shared similar risk-of-bias domains and methodological structure with the prospective cohorts. The meta-analysis was carried out on included 11 studies with 365 individuals who had replacement of their amalgam restorations with either a composite resin, gold, feldspathic ceramics or a combination of the three. Pooled Odds Ratio (OR) The meta-analysis produced a pooled OR of 122.18 (95 % CI: 55.64 to 268.26) with patients undergoing replacement of their amalgam restorations being 122 times more likely have lesion resolution than patients with no treatment. Heterogeneity (I^2^): Moderate heterogeneity was observed (I^2^ = 42 %), which justified the application of a random-effects model. This variation is attributed to differences in follow-up durations, lesion types, and replacement materials. The effect was statistically significant with p < 0.001, confirming a robust association between amalgam removal and the clinical recovery of OLLs (Fig. 4).

Although the pooled odds ratio was numerically very large, this estimate is consistent with the underlying study-level data, in which lesion resolution was frequent in replacement groups but rare in non-replacement comparators. In several cohorts, complete or partial healing occurred in the vast majority of patients after amalgam replacement, whereas spontaneous resolution in control or non-intervention arms was close to zero.

### 3.6 Subgroup and sensitivity analyses

Subgroup analyses further identified more information with respect to the primary outcome (Table 6). Lesion Type: Although erosive lesions showed a slightly stronger amelioration and resolution rate and were eligible to be included in the primary outcome, a statistically valid difference was not identified. Restorative Material: Gold and feldspathic ceramics had slightly more favourable outcomes than composite resin. Patch Testing: Lesions from patients that were positive with patch testing had a greater remission rate (83 %) than those lesions with negative/untried (58 %) patch testing. Geographic Region: Studies in Europe and Asia tended to have a better and more consistent remission rates. This may represent how most practitioners manage their patients with respect to their local practices and materials. Follow-Up: Studies with >12-month follow-up were more likely to achieve a maintainable state of remission of the lesion.

**Table 6:** Summary of subgroup outcomes.

Subgroup Category	Comparison Group	Resolution Rate (%)	Observations
Lesion Type	Erosive vs. Reticular	76 % vs. 68 %	Slightly better resolution in erosive type
Restorative Material	Gold/Ceramic vs. Composite	83 % vs. 71 %	Gold/ceramic favoured, but underpowered
Patch Test Result	Positive vs. Negative/Untested	83 % vs. 58 %	Predictive value evident
Geographic Region	Europe/Asia vs. Others	79 % vs. 61 %	Greater consistency in EU/Asia
Follow-Up Duration	≥12 months vs. <12 months	82 % vs. 66 %	More durable remission with longer follow-up

**Fig. 5:**
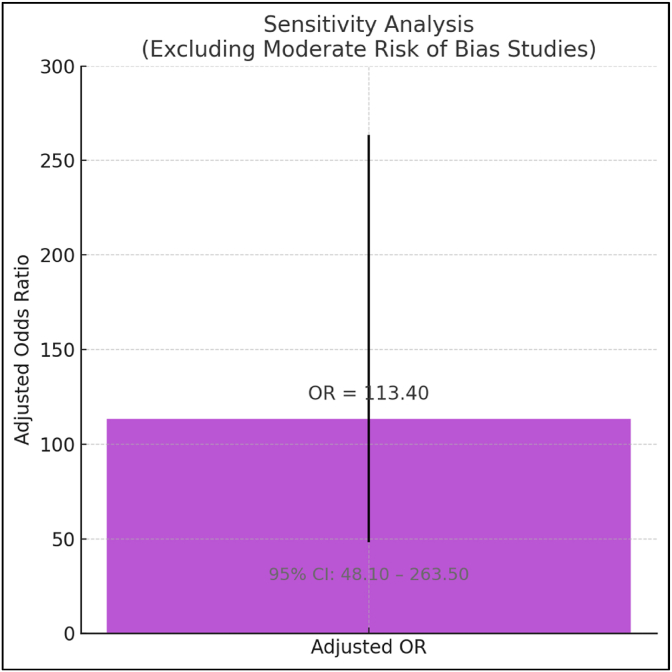
Sensitivity analysis of lesion remission outcomes after excluding studies with moderate risk of bias.

Sensitivity Analysis: Excluding studies with moderate risk of bias had essentially no effect on the effect size, providing additional reassurance regarding stability of findings (adjusted OR = 113.40, 95 % CI: 48.10–263.50) Fig. 5 represents the adjusted odds ratio (OR = 113.40) of complete lesion remission after excluding studies of moderate risk of bias. The error bars represent the 95 % confidence interval (CI: 48.10–263.50), demonstrating the robustness and stability of the treatment effect.

### 3.7 Publication bias and quality of evidence

**Fig. 6:**
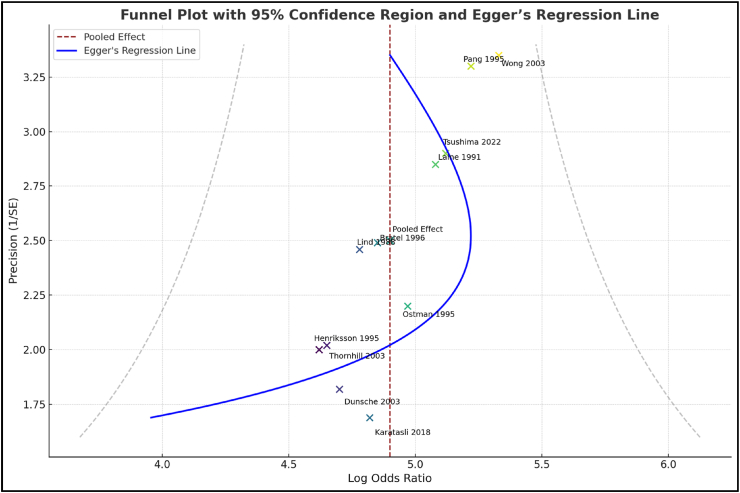
Funnel plot with 95 % confidence region (dotted funnel boundary) and Egger's regression line (solid blue). (For interpretation of the references to colour in this figure legend, the reader is referred to the Web version of this article.)

Visual exploration of the funnel plot (Fig. 6) indicated asymmetry that suggested publication bias and Egger's regression test was statistically significant (intercept of −2.29, p < 0.001), suggesting that studies with negative or null results were likely underreported and added inflation to the effect observed.

**Fig. 7:**
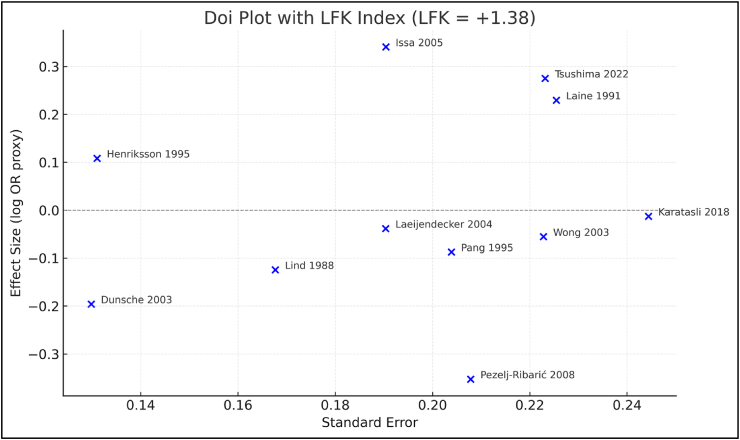
Doi plot with LFK index assessing small-study effects. Each point represents one included clinical study labelled by study name. The LFK index (+1.38) indicates minor asymmetry, a pattern expected in sparse-event binary meta-analyses.

To assess small study influence under sparse event situations more reliably, we constructed a Doi plot with LFK Index (Fig. 7) The Doi Plot indicates an LFK Value of 1.38 which represents a slight deviation from perfect symmetry, indicating a very slight asymmetric result. While this level of deviation may indicate an imbalance, it is not necessarily indicative of publication bias (a significant concern for meta-analysis) as it is common to have null or very low incident rates for comparator arms in meta-analysis studies. Crucially, in line with the funnel plot's visual impression, the overall dispersion of study effects stayed balanced around the pooled estimate. Thus, any small-study effects present are unlikely to have materially influenced the magnitude or direction of the overall effect.

The certainty of evidence for lesion resolution following amalgam replacement, as summarized in Table 7, was judged overall as low to moderate. The meta-analysis included all 11 observational studies (10 prospective non-randomized studies, 1 comparative observational cohort), all of which were evaluated for quality using the GRADE Tool. Due to the nature of the individual observational study designs being non-randomised, the evidence was initially rated as very low certainty with potential for Upgrade or Downgrade depending on Domain Specific Methodological characteristics. The potential for Downgrade was primarily because of the Risk of Bias due to Imbalances and Inconsistencies between Outcome Assessments, as well as the Risk of Imprecision due to Wide Ranges in Confidence Intervals due to Low Numbers of Patients in the Comparisons making it difficult to determine their significance/impact. Inconsistency was minimal, supported by overlapping confidence intervals and moderate heterogeneity, and indirectness concerns were low because all studies directly evaluated the target population, intervention, and clinical outcome. Although the Doi plot demonstrated only minor asymmetry (LFK = +1.38), this pattern is expected in sparse-event datasets and was not considered to meaningfully affect the certainty rating. The considerable size of the combined effect of upgrade evidence; the biological likelihood that the lesion will resolve once the restorative material causing the lesion has been removed; and that all the studies were in the same direction are the bases for the upgrade of this evidence. Overall, the convergence of all these considerations yields a level of certainty (Low-Medium) regarding the total body of work, since observational studies generally contain methodological limitations, yet the strength and consistency of the observed clinical effect outweighs the limitations inherent to these study designs.

**Table 7:** GRADE Summary of findings.

Outcome	Effect Estimate (OR, 95 % CI)	No. of Participants (Studies)	Certainty of Evidence	Comments
Lesion resolution after amalgam replacement	122.18 (55.64–268.26)	365 (11 studies)	●●●◯ Moderate	Downgraded for publication bias; strong association supports upgrade
Resolution with positive patch test	Higher vs. negative/untested	221 (7 studies)	●●●◯ Moderate	Patch testing useful but underreported and inconsistently interpreted
Gold/Ceramic vs. Composite	Slight increase in resolution	180 (5 studies)	●●◯◯ Low	Small sample sizes; needs further prospective validation
Long-term follow-up (≥12 mo)	Higher durability of remission	278 (8 studies)	●●●◯ Moderate	More stable outcomes in longer follow-up

## 4 Discussion

Our meta-analysis shows a very large association between amalgam replacement and clinical resolution of oral lichenoid lesions (pooled OR ≈122; 95 % CI ≈ 56–268), with moderate heterogeneity (I^2^ ≈ 42 %). The direction of effect is consistent across settings and materials, but variability in outcome definitions and follow-up likely contributes to dispersion; the magnitude should therefore be interpreted cautiously. Our findings complement the pathophysiologic distinction between OLL (contact-related, reversible after source removal) and OLP (idiopathic, autoimmune), already outlined in the Introduction, and should be interpreted within the current policy context that encourages phase-down of mercury-containing materials.

Patch-testing showed inconsistent diagnostic utility across studies. In our subgroup summaries, patch-positive patients tended to exhibit higher remission rates after amalgam replacement than patch-negative/untested patients, but estimates were limited by heterogeneous test panels, protocols, and reporting. Given these constraints—and the strong effect observed in lesions with a direct topographic contact to amalgam—clinical judgment anchored in lesion location and post-replacement response is more actionable than patch testing alone.

Of the 11 quantitative studies, 3 used any standardized Patient Reported Outcome Measures (PROMs) including the Visual Analogue Scale (VAS). Nevertheless, subjective symptom improvements, including burning, pain when eating and mucosal discomfort were noted in 7 of the studies. In many cases, patients reported symptom relief within 1–3 months of amalgam replacement, and prior to complete mucosal healing. The lack of use of PROMs is an important void in the literature. Since OLLs markedly and negatively affect oral function and quality of life, future trials should include validated instruments that assess patient-centered outcomes beyond visible healing (such as the Oral Health Impact Profile (OHIP), VAS, or Likert-based satisfaction scales).^53^

Issa et al.,^26^ noted resolution of lesions between 45 and 77 % after the replacement of amalgam restorations, particularly when restorations were in direct contact with the lesions. Additionally, Bratel et al.,^22^ reported an overall clinical benefit of 97.1 % with 29.5 % complete healing after restorative replacement in patients, despite other patients actively developing unfavorable patch test reactions; there were no lesions noted to have grown, which underscores that clinical topography should take precedence over patch test status. Pigatto et al.,^54^ found that there was 22 % complete clinical healing after lesions were removed and a significant correlation to lesion topography of the lesions removed and being positive on patch testing, but only 50 % of clinically healed lesions also healed histologically showing diners between clinical healing and histological healing. In Laine et al.,^19^ complete healing was 42 % in patients where lesions were patch test positive versus 20 % of the patients who were patch test negative, consistently suggesting that patients who were positive on patch testing could indicate an informed outcome but does not dictate their outcome. Crucially, Dunsche et al.,^23^ showed 59.7 % of lesions healed after removal compared to only 6.9 % in the controls, and in total, regardless of patch test status 97.1 % of patients experienced improvement underscoring that clinical topographic association is the strongest guide to modifications of restoration. Patch tests have limited value alone.

Although this meta-analysis shows that amalgam replacement is highly effective for OLLs, a subset of patients does not achieve full remission, underscoring the condition's multifactorial complexity and the need to clarify failure mechanisms. Topography is pivotal: unilateral lesions contiguous with an amalgam surface strongly suggest OLL,^55^ whereas bilateral or non-adjacent lesions raise the likelihood of OLP, where simple restorative replacement seldom yields resolution. Non-response may also reflect systemic autoimmune disease—for example, systemic lupus erythematosus, mucous membrane pemphigoid, or chronic graft-versus-host disease—which can mimic OLL/OLP clinically and typically do not resolve with amalgam removal because mucosal inflammation is sustained by immune dysregulation 56, 57, 58. In such cases, amalgam likely acts only as a secondary irritant, reinforcing the need for a biopsychosocial, interdisciplinary evaluation tailored to persistent or atypical presentations.^8^

The chronicity of lesions is an important prognostic factor. The longer lesions are present, the higher the likelihood of epithelial dysplasia, fibrosis, and keratinocyte apoptosis, rendering less reversible following removal of the offending contact.^59^^,^^60^ While early diagnosis and intervention have higher resolution rates,^61^ chronic lesions often have limited healing due to mucosal reorganization and epigenetic reprogramming of inflammatory pathways which emphasizes the need for early recognition and prompt replacement of amalgam to avoid irreversible change.

Psychosocial stressors, smoking, poor oral hygiene, and nutritional deficits (iron, folate, B12) contribute to chronic mucosal inflammation but are infrequently reported in clinical studies.^62^^,^^63^ Stress-related immunomodulation may alter cytokines and hinder healing even after allergen removal.^64^ Thus, persistent symptoms post-replacement warrant a biopsychosocial approach—behavioral counseling, dietary assessment, oral-hygiene reinforcement, and review of substituted materials (e.g., devices, stents, prostheses) for compatibility.^65^ Recovery is often gradual; several studies report improvement over 3–12 months, particularly for erosive and reticular lesions, underscoring the need for ≥6–12 months of follow-up in both research and practice to allow epithelial and immune homeostasis.^1^^,^^61^

Complete and meticulous amalgam removal is critical to outcomes: partial removal can leave alloy residues that continue to provoke mucosal immune responses.^66^ Healing is also contingent on the replacement material—biocompatible options (e.g., gold alloys, feldspathic porcelain, high-quality composite resin) can be more beneficial than low-grade options that may contribute irritation.^67^ There are also larger, more systemic considerations regarding the use of materials containing mercury beyond the dental office. The clarity of mercury's neurotoxicity and environmental concerns lend themselves to moving away from amalgam materials in general,^68^ but they are equally consistent with the phase-down of mercury-containing dental materials embodied in the Minamata Convention.^69^ Clinically, our findings align with this direction: in patients with suspected contact-related OLL, early, complete replacement combined with prudent medical co-management (e.g., tapering systemic corticosteroids when appropriate, targeted biopsy, and monitoring comorbid disease) is a responsible strategy that may reduce overall healthcare burden while improving patient outcomes.^2^^,^^8^

This review provides, to our knowledge, the first quantitative appraisal of lesion recovery after amalgam replacement in OLL, using comprehensive meta-analytic methods and explicit risk-of-bias assessment across diverse cohorts (continents, ages, lesion types), thereby enhancing generalizability. Key limitations include heterogeneous definitions of “recovery” (complete vs symptomatic improvement), variable follow-up and outcome documentation, limited blinding with potential observer bias, and evidence of publication bias on Egger's regression—each tempering precision and suggesting possible overestimation of effect. Clinically, clinicians should maintain an elevated index of suspicion for unilateral, contact-related lesions and view early, well performed replacement as an option of active care rather than a last resort. Policy-wise, the findings are consistent with phase-down strategies for mercury containing materials when suitable biocompatible alternatives are commercially available. Future work should prioritize prospective multicentre studies with standardized outcomes and follow-up, and explore biomarkers (genetic polymorphisms, cytokine profiles, T-cell reactivity) to refine patient selection and predict response.

Novel technologies in AI-based oral lesion monitoring and mobile diagnostic platforms will likely make it possible to track both lesion progression and response to treatment, providing timely insights and enhancing follow-up compliance. In the long term, development in oral pathology, restorative dentistry, and immunology will be pivotal for our understanding of mucosal hypersensitivity disorders. Interdisciplinary models must incorporate psychological assessment instruments also, as chronic oral lesions negatively affect quality of life, and mental health of an individual.

## 5 Conclusion

This meta-analysis provides clear evidence for the strong therapeutic effect for amalgam replacement in the management of oral lichenoid lesions (OLLSs) and confirms it to be a major treatment modality. The considerable odds ratio supports the clinical significance of such an approach and suggests that when the arrow is positioned as a focus on the amalgam replacement, it is good for patients and will minimize morbidity associated with these lesions. Given that amalgam restorations are actively used worldwide, the effects of these results for dental practice are numerous, suggesting an element of caution for clinicians to consider amalgam replacement in their patients with OLLs and greater efforts to develop evidence-based diagnostic protocols for at-risk patients.

## Patient/guardian consent

This was a systematic review and meta-analysis of existing studies, hence no patient or guardian consent was required.

## Author contributions

All the authors made a significant contribution to the work reported, whether that is in the conception, study design, execution, acquisition of data, analysis and interpretation, or all these areas, took part in drafting, revising, or critically reviewing the article; gave final approval of the version to be published; have agreed on the journal to which the article has been submitted; and agree to be accountable for all aspects of the work.

## PROSPERO registry number

The study protocol is registered with the International Prospective Register of Systematic Reviews (PROSPERO) - registration number CRDXXXXXXXXXX2.

## Ethical statement

This systematic review and meta-analysis used data from previously published studies and did not require ethical approval because no new data were gathered. The Institutional Ethics Committee thus waived ethical approval.

## Source(s) of support

The authors received collaboration funding from Airlangga Research Fund from Universitas Airlangga No 672/UN3/2024, 19 February 2024

## Availability of data and materials

All relevant data are within the manuscript and its Supporting Information files.

## Declaration of competing interest

The authors declare that they have no known competing financial interests or personal relationships that could have appeared to influence the work reported in this paper.
