# Supplementary material for: Clinical resolution of oral lichenoid lesions after amalgam replacement: A systematic review and meta-analysis of observational studies
Source: J Oral Biol Craniofac Res. 2026 Jan 28;16(2):101404. doi: 10.1016/j.jobcr.2026.01.008 (PMC12874582; doi:10.1016/j.jobcr.2026.01.008)
Supplement: Multimedia component 1 [file mmc1.docx]

# Supplementary Table S1.

# Database-specific Search Strategies, Coverage, and Yields

| Database | Platform / Interface | Coverage & Final Search Date | Verbatim Search Strategy | Records Retrieved |
| --- | --- | --- | --- | --- |
| PubMed/MEDLINE | NCBI | Inception–Dec 31, 2024 Final run: Dec 31, 2024 | ((((((("dental amalgam"[MeSH Terms] OR ("dental"[All Fields] AND "amalgam"[All Fields]) OR "dental amalgam"[All Fields] OR "amalgam"[All Fields]) AND restorations[All Fields]) OR (("mercury"[MeSH Terms] OR "mercury"[All Fields]) AND ("hypersensitivity"[MeSH Terms] OR "hypersensitivity"[All Fields] OR "allergy"[All Fields] OR "allergy and immunology"[MeSH Terms] OR ("allergy"[All Fields] AND "immunology"[All Fields]) OR "allergy and immunology"[All Fields]))) AND (("mouth"[MeSH Terms] OR "mouth"[All Fields] OR "oral"[All Fields]) AND lichenoid[All Fields] AND lesions[All Fields])) OR ("lichen planus, oral"[MeSH Terms] OR ("lichen"[All Fields] AND "planus"[All Fields] AND "oral"[All Fields]) OR "oral lichen planus"[All Fields] OR ("oral"[All Fields] AND "lichen"[All Fields] AND "planus"[All Fields]))) OR ("dermatitis, allergic contact"[MeSH Terms] OR ("dermatitis"[All Fields] AND "allergic"[All Fields] AND "contact"[All Fields]) OR "allergic contact dermatitis"[All Fields] OR ("contact"[All Fields] AND "allergy"[All Fields]) OR "contact allergy"[All Fields])) AND (((((("dental amalgam"[MeSH Terms] OR ("dental"[All Fields] AND "amalgam"[All Fields]) OR "dental amalgam"[All Fields] OR "amalgam"[All Fields]) AND restoration[All Fields] OR "replacement"[All Fields])) OR (composite[All Fields] AND restorations[All Fields])) OR (("gold"[MeSH Terms] OR "gold"[All Fields]) AND restorations[All Fields])) OR ("glass ionomer cements"[MeSH Terms] OR ("glass"[All Fields] AND "ionomer"[All Fields] AND "cements"[All Fields]) OR "glass ionomer cements"[All Fields] OR ("glass"[All Fields] AND "ionomer"[All Fields] AND "cement"[All Fields]) OR "glass ionomer cement"[All Fields])) OR ("dental porcelain"[MeSH Terms] OR ("dental"[All Fields] AND "porcelain"[All Fields]) OR "dental porcelain"[All Fields] OR "porcelain"[All Fields]))) AND (("wound healing"[MeSH Terms] OR ("wound"[All Fields] AND "healing"[All Fields]) OR "wound healing"[All Fields] OR "healing"[All Fields]) AND lichenoid[All Fields] AND lesions[All Fields]) | 62 |
| EMBASE | Elsevier/Ovid | Inception–Dec 31, 2024 Final run: Dec 31, 2024 | ('oral lichenoid lesion'/exp OR 'oral lichenoid lesion':ti,ab OR (lichenoid:ti,ab AND (oral:ti,ab OR mouth:ti,ab)) OR 'oral lichen planus'/exp OR 'contact dermatitis'/exp) AND ('dental amalgam'/exp OR 'dental amalgam':ti,ab OR amalgam:ti,ab) AND (replace*:ti,ab OR replacement:ti,ab OR restoration*:ti,ab OR 'composite resin'/exp OR 'glass ionomer cement'/exp OR (gold:de AND restoration*:ti,ab) OR porcelain:ti,ab OR ceramic*:ti,ab) AND (heal*:ti,ab OR resolution:ti,ab OR improve*:ti,ab OR 'wound healing'/exp) AND [humans]/lim | 520 |
| CENTRAL | Cochrane Library | Inception–Dec 31, 2024 Final run: Dec 31, 2024 | (("oral lichenoid" OR "oral lichen planus" OR (lichenoid AND oral)) AND (amalgam) AND (replace* OR removal OR restoration) AND (heal* OR resolution OR improvement)) in Trials | 38 |
| CINAHL | EBSCOhost | Inception–Dec 31, 2024 Final run: Dec 31, 2024 | (MH "Oral Lichen Planus" OR TI ("oral lichenoid" OR "oral lichen planus") OR AB ("oral lichenoid" OR "oral lichen planus")) AND (MH "Dental Amalgam" OR TI (amalgam) OR AB (amalgam)) AND (TI (replace* OR removal OR restoration) OR AB (replace* OR removal OR restoration)) AND (TI (heal* OR resolution OR improvement) OR AB (heal* OR resolution OR improvement)) | 90 |
| Scopus | Elsevier | Inception–Dec 31, 2024 Final run: Dec 31, 2024 | TITLE-ABS-KEY(("oral lichenoid" OR "oral lichen planus" OR (lichenoid AND oral)) AND (amalgam) AND (replace* OR removal OR restoration) AND (heal* OR resolution OR improvement)) AND (LIMIT-TO(DOCTYPE,"ar") OR LIMIT-TO(DOCTYPE,"re")) AND (EXCLUDE(PUBYEAR,2025)) | 1100 |
| PsycINFO | APA/EBSCOhost | Inception–Dec 31, 2024 Final run: Dec 31, 2024 | (TI ("oral lichenoid" OR "oral lichen planus") OR AB ("oral lichenoid" OR "oral lichen planus")) AND (TI (amalgam) OR AB (amalgam)) AND (TI (replace* OR removal) OR AB (replace* OR removal)) | 30 |
| ERIC | EBSCOhost | Inception–Dec 31, 2024 Final run: Dec 31, 2024 | (TI ("oral lichenoid" OR "oral lichen planus") OR AB ("oral lichenoid" OR "oral lichen planus")) AND (amalgam) AND (replace* OR removal) | 20 |
| ScienceDirect | Elsevier | Inception–Dec 31, 2024 Final run: Dec 31, 2024 | ("oral lichenoid" OR "oral lichen planus") AND amalgam AND (replacement OR removal) AND (healing OR resolution OR improvement) [All fields; filtered to Research/Review Articles] | 210 |
| Additional Sources (manual searching, citation tracking, and reference list screening) | Not Applicable | Inception–Dec 31, 2024 Final run: Dec 31, 2024 | Not applicable | 298 |
| Total Records |  |  |  | 2,368 |
